# Supplementary figures and images for: Early immune response against Fonsecaea pedrosoi requires Dectin-2-mediated Th17 activity, whereas Th1 response, aided by Treg cells, is crucial for fungal clearance in later stage of experimental chromoblastomycosis
Source: PLoS Negl Trop Dis. 2020 Jun 15;14(6):e0008386. doi: 10.1371/journal.pntd.0008386 (PMC7316354; doi:10.1371/journal.pntd.0008386)

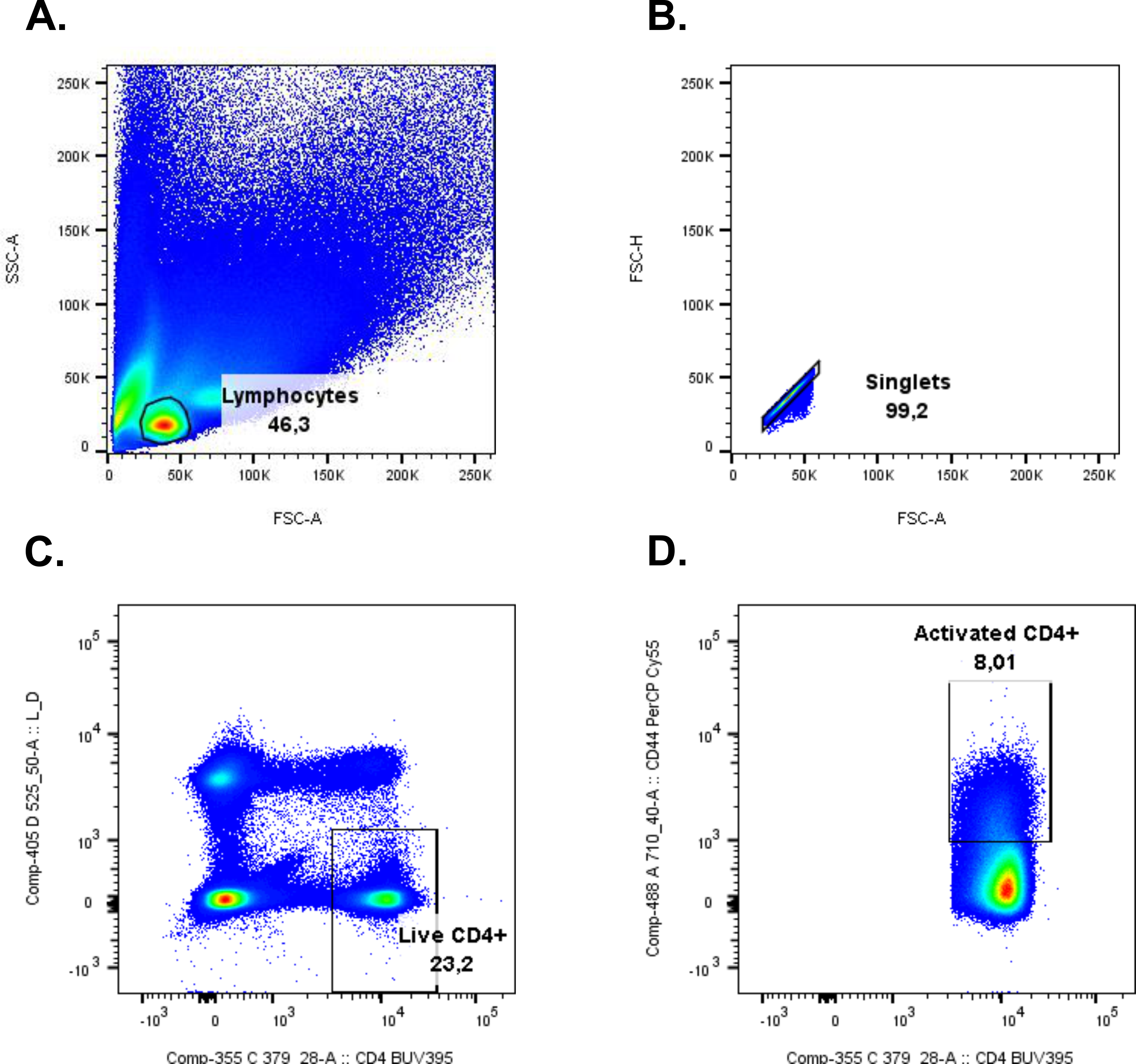

Supplement: S1 Fig — In order to select a relevant T lymphocyte population for proper investigation, a gate strategy was built, aiming to select only lymphocytes, according to their morphometric aspects (A), which were isolated (B), live (C) and activated (D). Live CD4+ was established using Live and Dead dye, and activated CD4+ cells were then gated considering a hi expression of CD44. (TIF) [file pntd.0008386.s002.tif]

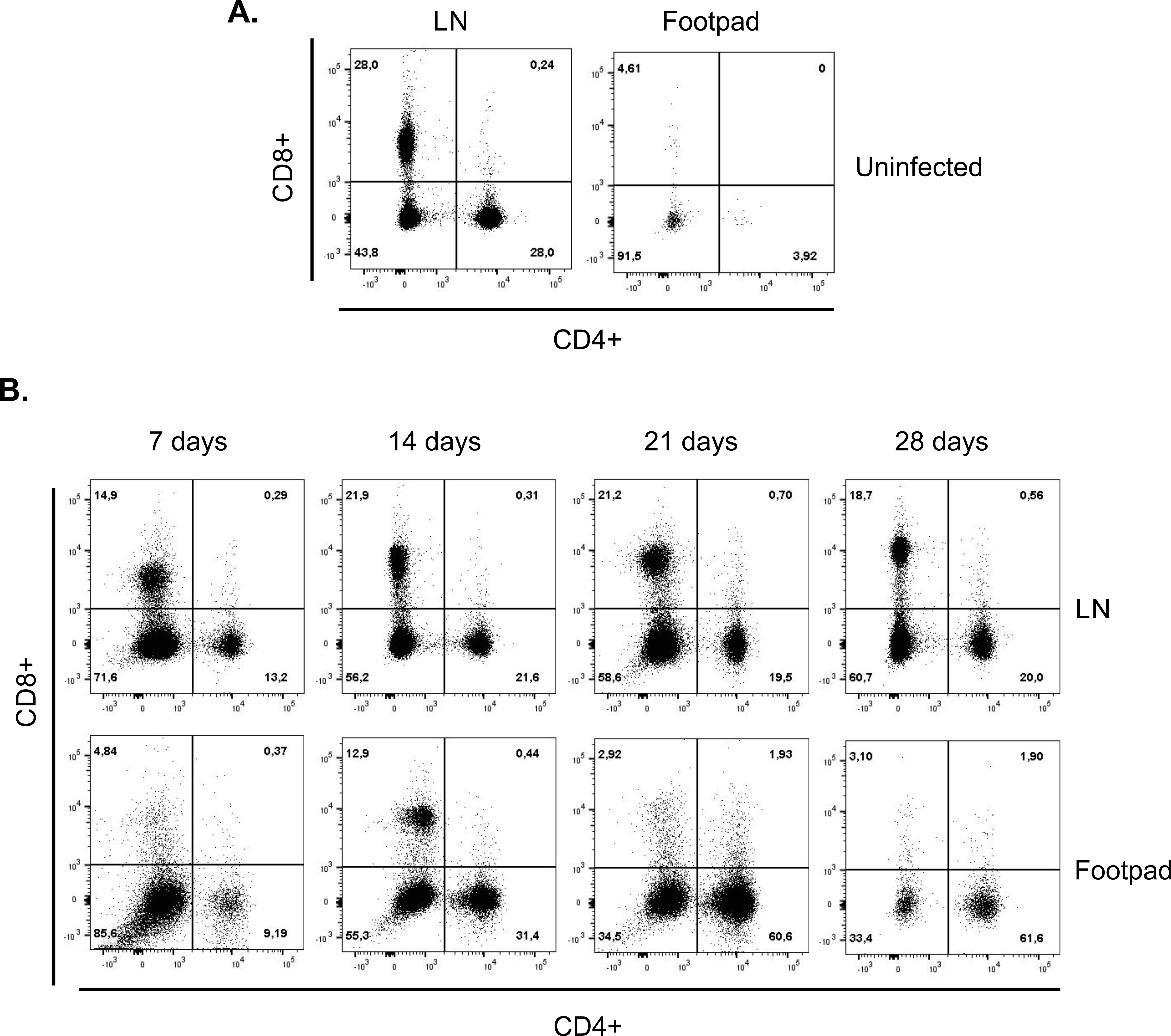

Supplement: S2 Fig — Cytometry dotplots aimed to identify and quantify CD8+ and CD4+ T cells in the footpad and draining lymph node (LN) in the course of experimental CBM (B). Uninfected animals were used as control (A). (TIF) [file pntd.0008386.s003.tif]

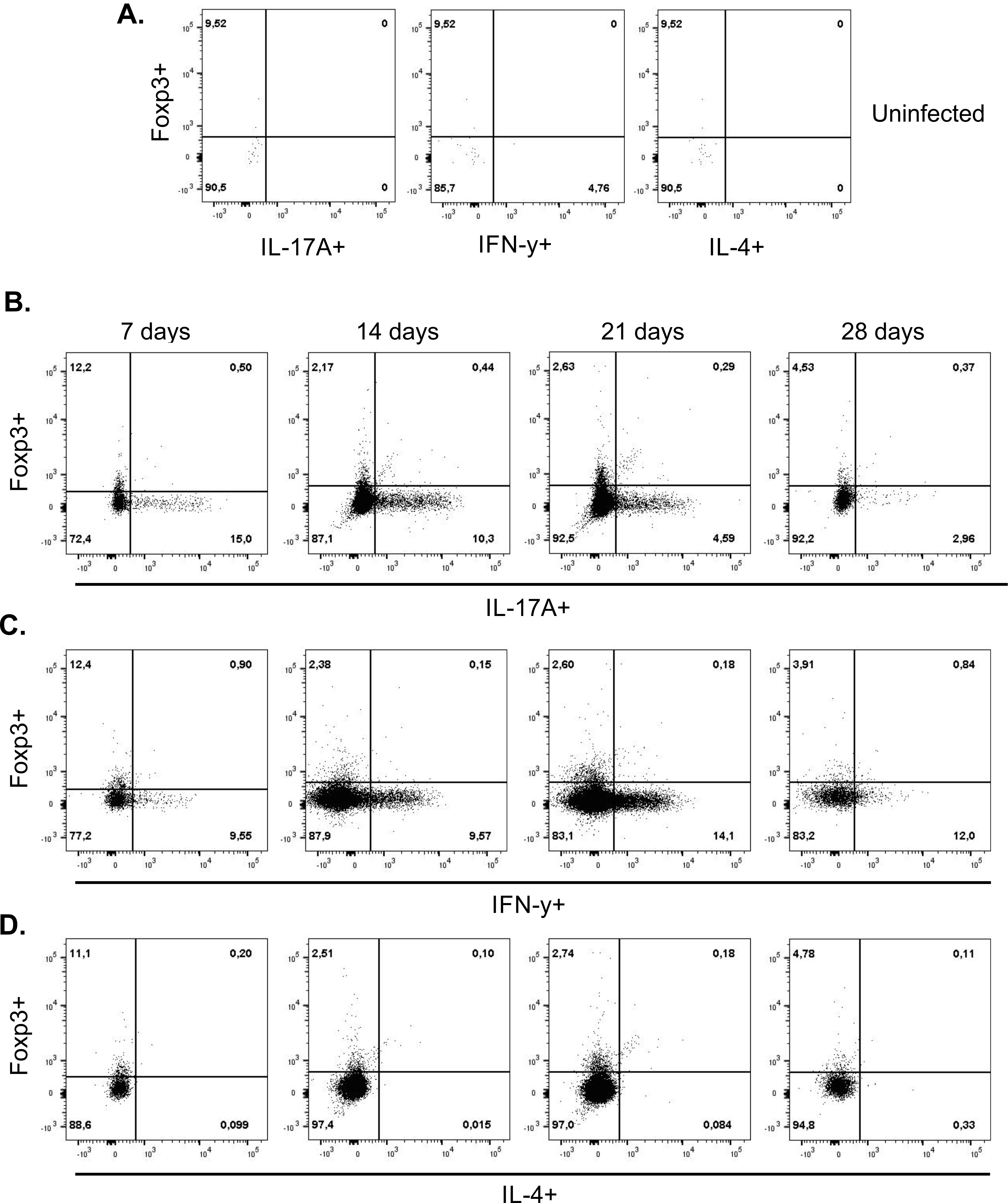

Supplement: S3 Fig — Cytometry dotplots aimed to identify Foxp3+, IL-17A+, IFN-Y+, and IL-4+ CD4+ T cells’ subpopulation in the footpad of animals infected with F. pedrosoi in the course of experimental CBM (B). Uninfected animals were used as control (A). (TIF) [file pntd.0008386.s004.tif]

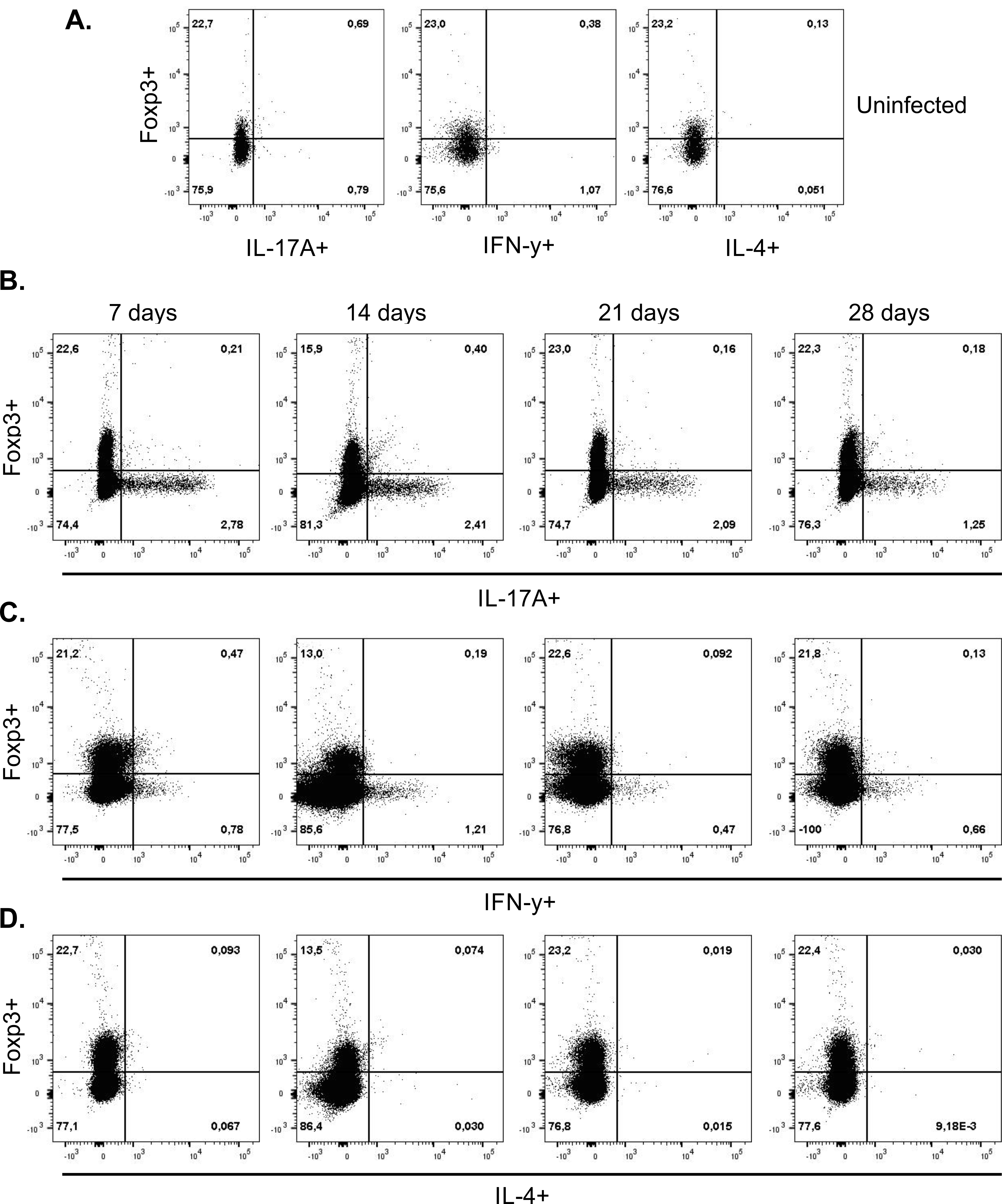

Supplement: S4 Fig — Cytometry dotplots aimed to identify Foxp3+, IL-17A+, IFN-Y+, and IL-4+ CD4+ T cells’ subpopulation in draining lymph node (LN) in the course of experimental CBM (B). Uninfected animals were used as control (A). (TIF) [file pntd.0008386.s005.tif]

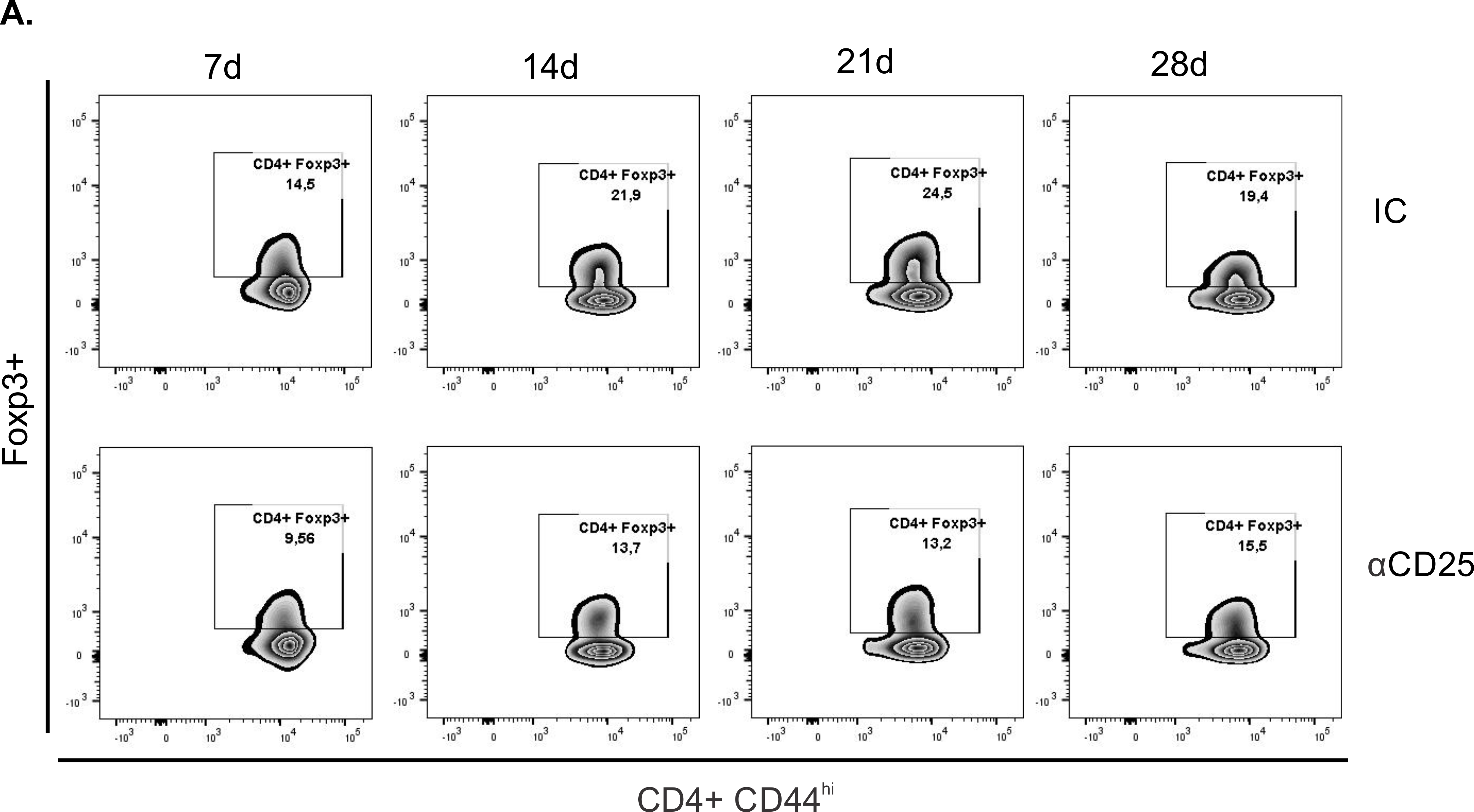

Supplement: S5 Fig — (TIF) [file pntd.0008386.s006.tif]

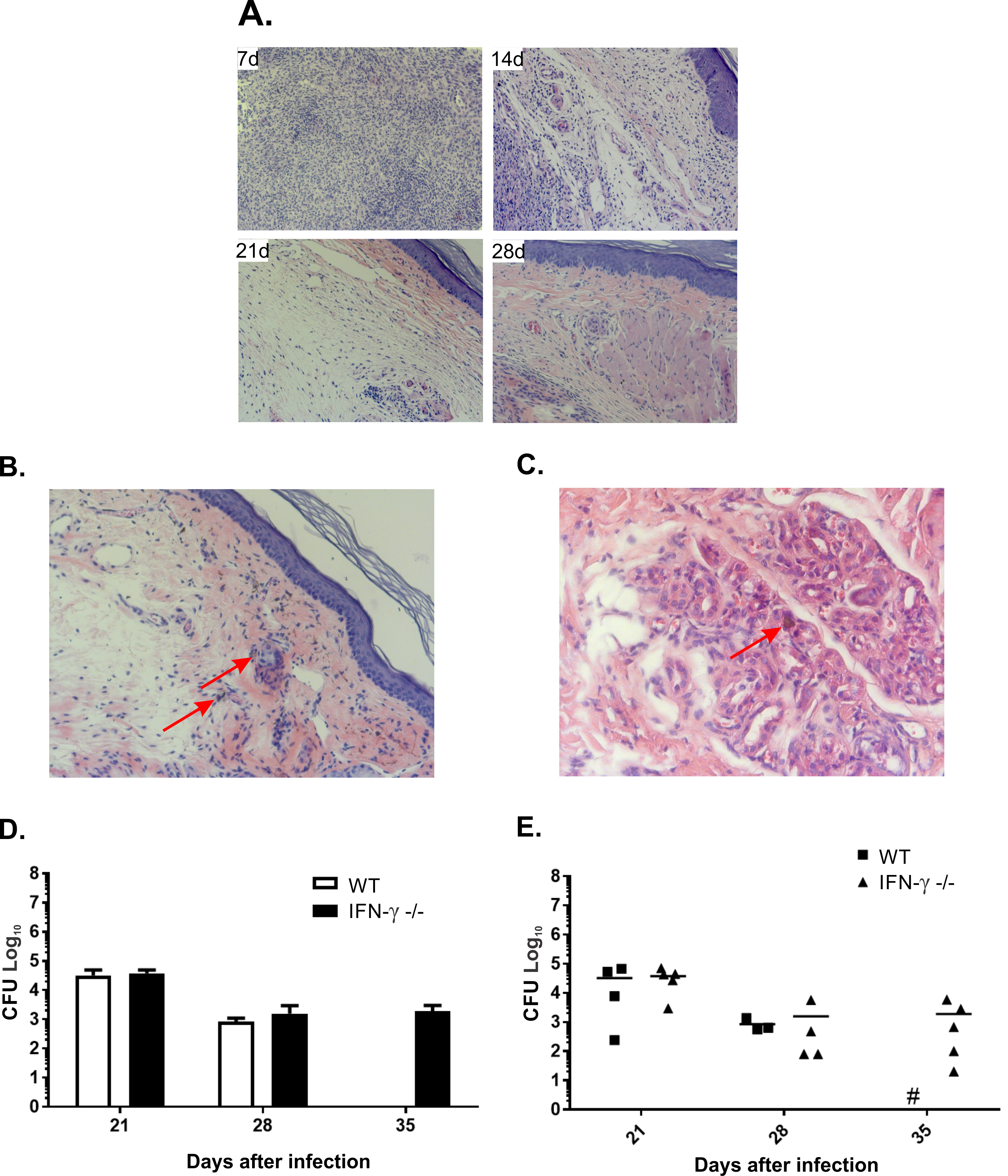

Supplement: S6 Fig — Histopathology of animals treated with isotype control and used as a control group for inflammation level measures, HE staining and 200x magnification (A). Histopathology of animals treated with αIFN-γ after 28 days of infection is displayed, showing the presence of muriform cells (arrows) in 200x (B) and 400x magnification (C). CFU quantification in IFN-γ -/- animals shows impaired fungal clearance after 28 and 35 days of infection (D-E). (TIF) [file pntd.0008386.s007.tif]

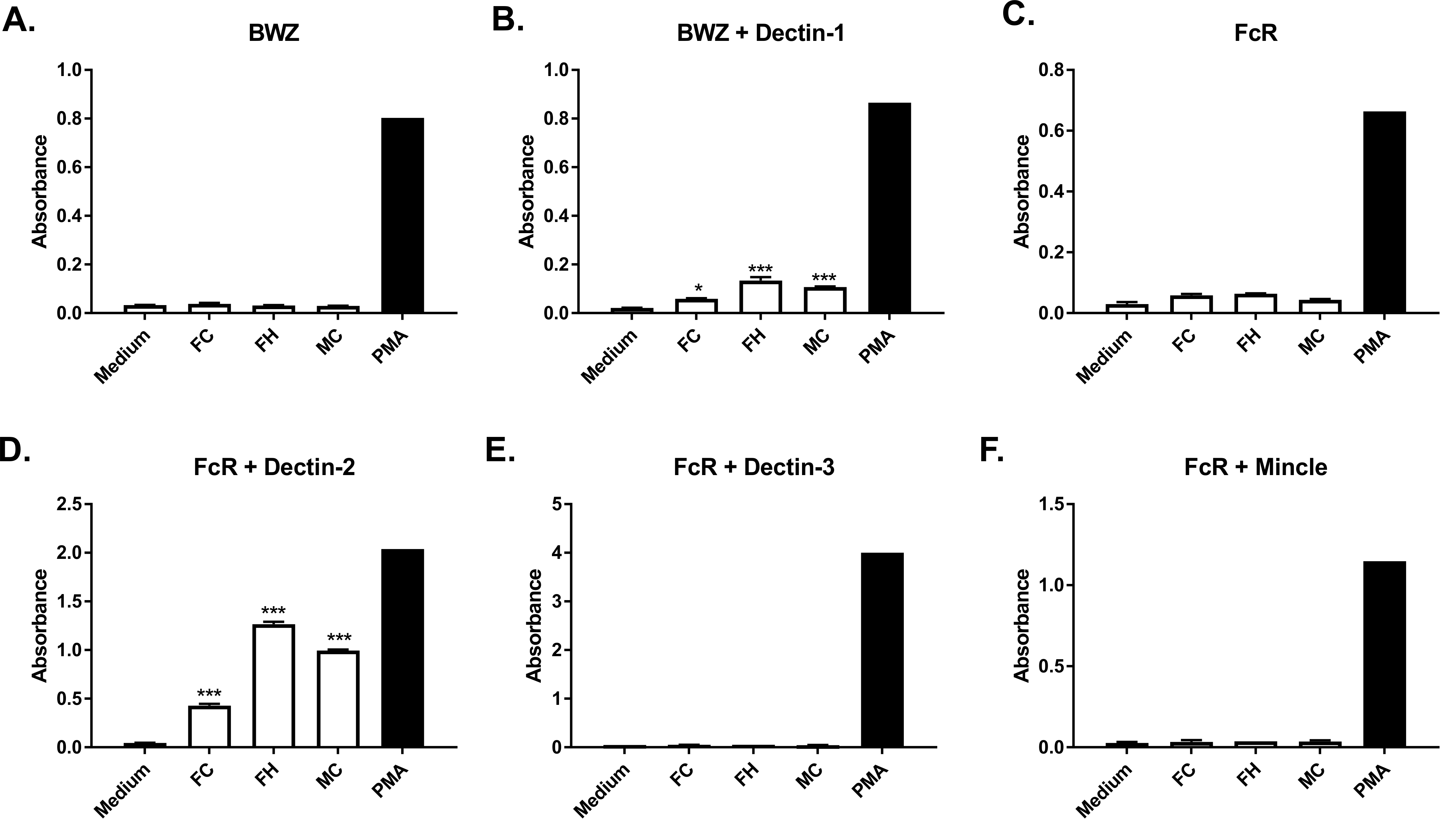

Supplement: S7 Fig — Interaction test between fungal forms with reporter cells expressing dectin-1 (B), dectin-2 (D), dectin-3 (E) and mincle (F) and carrying NFAT-lacZ construct was evaluated. Cells not expressing CRL (A) or expressing only FcR (C) were used as controls. * P <0.05 and *** P <0.001. (TIF) [file pntd.0008386.s008.tif]
