# Supplementary material for: Early immune response against Fonsecaea pedrosoi requires Dectin-2-mediated Th17 activity, whereas Th1 response, aided by Treg cells, is crucial for fungal clearance in later stage of experimental chromoblastomycosis
Source: PLoS Negl Trop Dis. 2020 Jun 15;14(6):e0008386. doi: 10.1371/journal.pntd.0008386 (PMC7316354; doi:10.1371/journal.pntd.0008386)
Supplement: Attachment — Submitted filename: Responses to the reviews.doc [file pntd.0008386.s009.doc]

Brasilia, April 14^th^, 2020

Dear

Mr. Todd B. Reynolds

Deputy Editor

First of all, we would like to thank for referees’ comments, which have improved the manuscript quality. All recommendations raised by the two reviewers were taken into consider, so that we have altered the manuscript text to match the reviewer’s counsel. Also, we have answered point-by-point the reviewer’s comments.

According to PACE report all figures meet PLOS requirements, so that they can be published in the highest quality.

In accordance with data requirements, the numerical data used in all figures are included in Data S1.

We hope the revised manuscript is now suitable for publication in PLOS Neglected Tropical Diseases.

I am looking forward to hearing from you

Yours sincerely,

Anamelia Lorenzetti Bocca, PhD

Lab. Applied Immunology – Institute of Biological Sciences

University of Brasília - Brazil

FAX: (+55 61) 33073056

e-mail: [albocca@unb.br](mailto:albocca@unb.br)

**ANSWERS OF REVIEWERS COMMENTS**

Lines references were made according to the clean version of the revised article.

**Methods**

*Reviewer #1:* *(…) The unique question is about ethical requirements concerning to the animal ethical committee permission. In case it was done, I suggest to present it in the methodology session.*

**Answer:** as described in lines 192-199, in the methodology session, all procedures involving animals carried out in the USA were approved by the Animal Care Committee of the University of Wisconsin – Madison (Protocol n.^o^ M00969) and procedures carried out in Brazil were approved by the Ethics Committee for Scientific Studies of the University of Brasilia (UnBDoc n.^o^ 135976/2014 and protocol n.º 46/2017). There was no additional requirement than those already incorporated in the methodology session.

**Conclusions**

*Reviewer #1: (…) The unique concern is why the authors didn't test the related species that are also considered causal agents of the disease? Such as, e.g., F. monophora and or Cladophialophora carrionii? Even though other species were not included in the study, I suggest to have at least one a paragraph in the discussion session about the disease clinic variations, if is it exclusively caused by the host's responses or not? A paragraph about this issue would be interesting, specially, as future perspective of this work in order to test other species and clarify more the immunology profile of this disease.*

**Answer:** In fact, chromoblastomycosis has a multietiological nature, so that several dimorphic fungi are related to CBM etiology. *Fonsecaea pedrosoi* and *Cladophialophora carrionii* are the main species identified in patients with the disease. They share phenotypical characteristics such as the ability to change their cells into muriform cells in infected tissue. CBM can also be caused by *Fonsecaea monophora*, *Fonsecaea compacta*, *Phialophora verrucosa*, *Rhinocladiella aquaspersa* or *Exophiala dermatitidis* [1–6]. *Exophiala jeanselmei* and *Exophiala spinifera* have also been observed to form muriform cells in typical CBM lesions. Molecular taxonomic studies have revealed that *F. compacta* represents a morphological variant of *F. pedrosoi* and, recently, *F. monophora* has been described as a new species with a phenotype similar to *F. pedrosoi*, however, genetically distinct [7–9].

Although highly relevant, it was not possible to extend the scope of this work to include other etiologic agents as it would significantly increase the magnitude of the work, supplanting the means available to its conduction.

Recent studies have shown that the different etiological agents of CBM activate immune cells in a similar way leading to resembling immune responses [10–13]. However, it is reasonable to think that the disease is the result of the unique interaction between the host and pathogen, so that further studies should be conducted in order to confirm whether findings described in the present work also apply to other CBM etiological agents.

Therefore, as suggested by reviewer 1, a paragraph was added to the discussion session addressing this issue (lines 521-525).

*Reviewer #1: (…) The other issue is that the authors inoculated the animals with muriforms cells produced in vitro. Do the authors have previous experience or tested on differences about response using fungal propagules for inoculation instead of muriform cells? If yes could you please discuss it?*

**Answer:** as described in lines 180-183 in methods section, animals were inoculated with fungal propagules and not with muriform cells. Purified muriform cells were applied in reporter cell assay. In our previews work we describe the evolution of murine CBM after infection with different fungal forms, such as conidia, hyphae and muriform cells. Inoculation with hyphae, muriform cells and fungal propagules were able to establish a murine CBM with skin lesions and histopathological aspects similar to that found in humans, so that infection with only muriform cells induced intense inflammatory response and delayed the healing process observed in mice [14]. Infection with fungal propagules (containing hyphae and conidia) mimics what happens in nature so that host is infected mostly with hyphae and conidia [15]. Although it is possible, muriform cells are rarely found in environmental conditions outside the host.

**Editorial and Data Presentation Modifications**

*Reviewer #1:*

*1- Authors should review the abbreviation of the volume unit used and standardize it. For example, throughout the text the authors used L and l for liter ( see e.g. the line 160 that was used mL and μl). I suggest using L for liters (e.g. μL and mL) and "l"only for chemical formulas;*

**Answer:** The paper was proofread carefully in order meet reviewer suggestion.

*3- In the methodology is necessary to provide the register number of the approval from the animals ethics committee.*

**Answer:** Animals ethics committee approval numbers were added in methods section, as shown in line 192-199.

*4- The authors must do a review of the scientific names, which must be in italics in all sections of the manuscript. In the reference section there are several scientific names that are not in Italic.*

**Answer:** The paper was proofread carefully in order to apply italic to all scientific names.

*5- I suggest to have at least one paragraph in the discussion session about the disease clinic variations, if is it exclusively caused by the host's responses or not? A paragraph about this issue would be interesting, specially, as future perspective of this work, in order to test other species and clarify more about the immunology profile of this disease.*

**Answer:** A new paragraph was added addressing these issue. See lines 521-525.

*Reviewer #2: Discussion needs to be broadened based on recent literature advances on the subject.*

**Answer:** Discussion was broadened as suggested.

**Summary and General Comments**

*Reviewer #1:* *(...) In general it is a relevant article and it is well written with only minor suggested corrections and some questions concerning to the strains tested and inoculum form that could be stressed on the discussion session.*

**Answer:** as mentioned above, animals were inoculated with fungal propagules of *F. pedrosoi* mimicking natural process of infection in nature.

*Reviewer #2: (…) More discussion about the relation between the author's findings on TH1 and TH17 and the reference:*

*Sousa, M., Reid, D., Schweighoffer, E., Tybulewicz, V., Ruland, J., Langhorne, J., Yamasaki, S., Taylor, P., Almeida, S., Brown, G. (2011). Restoration of Pattern Recognition Receptor Costimulation to Treat Chromoblastomycosis, a Chronic Fungal Infection of the Skin Cell Host & Microbe 9(5), 436-443.* [*https://dx.doi.org/10.1016/j.chom.2011.04.005*](https://dx.doi.org/10.1016/j.chom.2011.04.005)

*Sousa, M., Belda, W., Spina, R., Lota, P., Valente, N., Brown, G., Criado, P., Benard, G. (2014). Topical Application of Imiquimod as a Treatment for Chromoblastomycosis Clinical Infectious Diseases 58(12), 1734-1737.* [*https://dx.doi.org/10.1093/cid/ciu168*](https://dx.doi.org/10.1093/cid/ciu168)

*Where the activation of some PRR via TLR ligands or imiquimod was able to help curing the disease.*

**Answer**: Discussion session was improved and broadened to address those issues. As can be seen in lines 463-467, 476-478, 483-488.

**References**

1.	Borelli D. [Acrotheca aquaspersa nova, new species agent of chromomycosis]. Acta científica Venez [Internet]. 1972;23(6):193–6. Available from: http://www.ncbi.nlm.nih.gov/pubmed/4677524

2.	Gruber B, Rippon JW, Dayal VS. Phaeomycotic cyst (chromoblastomycosis) of the neck. Arch Otolaryngol Head Neck Surg [Internet]. 1988 Sep;114(9):1031–2. Available from: http://www.ncbi.nlm.nih.gov/pubmed/3408571

3.	McGinnis MR. Chromoblastomycosis and phaeohyphomycosis: new concepts, diagnosis, and mycology. J Am Acad Dermatol [Internet]. 1983 Jan [cited 2012 Sep 18];8(1):1–16. Available from: http://www.ncbi.nlm.nih.gov/pubmed/6826791

4.	Matsumoto T, Matsuda T, McGinnis MR, Ajello L. Clinical and mycological spectra of Wangiella dermatitidis infections. Mycoses [Internet]. 36(5–6):145–55. Available from: http://www.ncbi.nlm.nih.gov/pubmed/8264710

5.	López Martínez R, Méndez Tovar LJ. Chromoblastomycosis. Clin Dermatol [Internet]. 2007 [cited 2012 Jul 19];25(2):188–94. Available from: http://www.ncbi.nlm.nih.gov/pubmed/17350498

6.	Ameen M. Chromoblastomycosis: clinical presentation and management. Clin Exp Dermatol [Internet]. 2009 Dec [cited 2012 Jul 19];34(8):849–54. Available from: http://www.ncbi.nlm.nih.gov/pubmed/19575735

7.	Naka W, Harada T, Nishikawa T, Fukushiro R. A case of chromoblastomycosis: with special reference to the mycology of the isolated Exophiala jeanselmei. Mykosen [Internet]. 1986 Oct;29(10):445–52. Available from: http://www.ncbi.nlm.nih.gov/pubmed/3540662

8.	Padhye AA, Ajello L. A case of chromoblastomycosis with special reference to the mycology of the isolated Exophiala jeanselmei. Mykosen [Internet]. 1987 Mar;30(3):134. Available from: http://www.ncbi.nlm.nih.gov/pubmed/3587275

9.	Barba-Gómez JF, Mayorga J, McGinnis MR, González-Mendoza A. Chromoblastomycosis caused by Exophiala spinifera. J Am Acad Dermatol [Internet]. 1992 Feb;26(2 Pt 2):367–70. Available from: http://www.ncbi.nlm.nih.gov/pubmed/1569261

10.	Wu W, Zhang R, Wang X, Song Y, Li R. Subcutaneous infection with dematiaceous fungi in Card9 knockout mice reveals association of impair neutrophils and Th cell response. J Dermatol Sci [Internet]. 2018 Nov;92(2):215–8. Available from: https://linkinghub.elsevier.com/retrieve/pii/S0923181118303074

11.	Da Glória Sousa M, Reid DM, Schweighoffer E, Tybulewicz V, Ruland J, Langhorne J, et al. Restoration of pattern recognition receptor costimulation to treat chromoblastomycosis, a chronic fungal infection of the skin. Cell Host Microbe. 2011;9(5):436–43.

12.	Wevers B a., Kaptein TM, Zijlstra-Willems EM, Theelen B, Boekhout T, Geijtenbeek TBH, et al. Fungal engagement of the C-type lectin mincle suppresses dectin-1-induced antifungal immunity. Cell Host Microbe [Internet]. Elsevier; 2014;15(4):494–505. Available from: http://dx.doi.org/10.1016/j.chom.2014.03.008

13.	Wüthrich M, Wang H, Li M, Lerksuthirat T. F . pedrosoi -induced Th17-cell differentiation in mice is fostered by Dectin-2 and suppressed by Mincle recognition. 2015;1–22.

14.	Siqueira IM, Castro R, Leonhardt L, Miranda, Nishibe C, Almeida N, Tavares AH, et al. Modulation of the immune response by Fonsecaea pedrosoi morphotypes in the course of experimental chromoblastomycosis and their role on inflammatory response chronicity. 2017;1–23.

15.	Esterre P, Queiroz-Telles F. Management of chromoblastomycosis: novel perspectives. Curr Opin Infect Dis [Internet]. 2006 Apr;19(2):148–52. Available from: http://www.ncbi.nlm.nih.gov/pubmed/16514339
